# History of exposure to copper influences transgenerational gene expression responses in *Daphnia magna*

**DOI:** 10.1080/15592294.2023.2296275

**Published:** 2023-12-28

**Authors:** Guilherme Jeremias, Ana-Belén Muñiz-González, Fernando José Mendes Gonçalves, José-Luis Martínez-Guitarte, Jana Asselman, Joana Luísa Pereira

**Affiliations:** a CESAM - Centre for Environmental and Marine Studies & Department of Biology, University of Aveiro, Aveiro, Portugal; b Biology & Toxicology Group, Department of Mathematics, Physics, and Fluids, National Distance Education University (UNED), Madrid, Spain; c Blue Growth Research Lab, Ghent University, Ostend, Belgium

**Keywords:** Metals, invertebrates, molecular responses, RT-PCR, transgenerational effects, epigenetics

## Abstract

The establishment of transgenerational effects following chemical exposure is a powerful phenomenon, capable of modulating ecosystem health beyond exposure periods. This study assessed the transgenerational effects occurring due to copper exposure in the invertebrate *D. magna* at the transcriptional level, while evaluating the role of exposure history on such responses. Thus, daphnids acclimated for several generations in a copper vs. clean medium were then exposed for one generation (F0) to this metal, and monitored for the following non-exposed generations (F1, F2 and F3). Organisms differing in exposure histories showed remarkably different transcriptional profiles at the F0, with naïve organisms being more profoundly affected. These trends were confirmed for F3 treatments, which presented different transcriptional patterns for genes involved in detoxification, oxidative stress, DNA damage repair, circadian clock functioning and epigenetic regulation. Furthermore, regardless of exposure history, a great number of histone modifier genes were always found transcriptionally altered, thus suggesting the involvement of histone modifications in the response of *Daphnia* to metal exposure. Lastly, remarkably distinct transgenerational transcriptional responses were found between naïve and non-naïve organisms, thereby highlighting the influence of exposure history on gene expression and confirming the capacity of metals to determine transgenerational transcriptional effects across non-exposed generations.

## GRAPHICAL ABSTRACT

**Figure:**
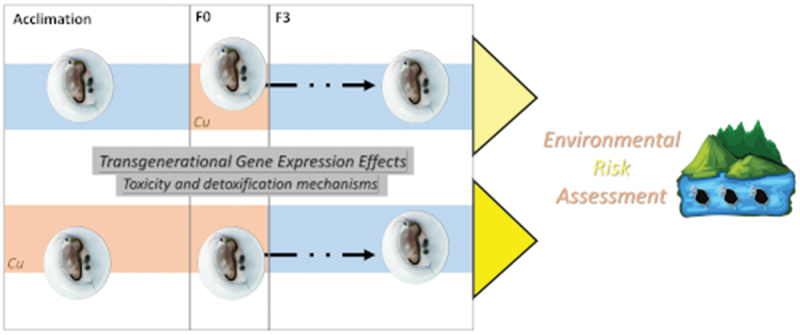

## Introduction

Freshwaters are vital for humanity by providing a direct source for water consumption and supporting many important natural systems for human well-being [1]. Expanding human activities determined profound changes in the physical and chemical properties of freshwater systems, and as a result, freshwater biodiversity has been declining at a higher rate than their terrestrial and marine counterparts [2,3]. Particularly, the increase of chemical inputs to freshwaters has been noticed worldwide, including the discharge of metal-burdened effluents originating from agricultural and urban areas, mining and other industries [1,4]. Although some metals are essential, at high concentrations these are toxic to freshwater biodiversity, while also presenting bioaccumulation potential [5,6]. Therefore, the effective protection of freshwater ecosystems calls for the definition of accurate regulatory benchmarks, which require a detailed understanding of the toxic potential and mechanisms of toxic action of metals [4,7].

The response of an organism to a chemical exposure is largely influenced by the characteristics of exposure, including its magnitude, frequency and duration [8,9]. Furthermore, it has been uncovered that past exposure to chemicals can shape the stress responses and life-histories of newly exposed organisms [10–12]. Historic exposure to metals critically influences the current stress tolerance of freshwater organisms; yet, the evidence is contradictory, with some studies showing that those organisms facing historic metal contamination presented a better capacity to regulate key molecular mechanisms conferring stress resistance, while others finding that the most experienced genotypes presented reduced gene diversity at detoxification genes, which seemed to underpin their lower tolerance to new exposures [10,13,14]. However, most of this literature has focused on the evolutionary direction followed by different genotypes and clones under a given stress scenario, and their potential constrains on a future response [10,13,15]. Alternatively, the analysis of gene expression patterns originating from a specific genotype could be advantageous by allowing the interpretation of life-history effects across several generations without the confounding effect of genetic diversity [16–18].

Despite the need for future clarification of this matter, it is apparent that sometimes the effects induced by chemicals only express themselves a posteriori [9,19,20]. Following this lead, an increasing body of ecotoxicological and risk assessment literature has been focusing on the transgenerational effects resulting from exposure to stressors [21,22]. This made clear that chemicals can induce long-lasting biological effects, thus extending their influence beyond originally exposed generations, i.e., affecting subsequent non-exposed generations [23,24]. Such chemical-induced transgenerational effects can prompt phenotypic changes that have either positive or negative fitness consequences, thus driving population and evolutionary change [25–27]. Still, the prevalence of transgenerational effects is excluded from the frameworks for the environmental risk assessment of chemicals, which are mostly based on conventional experiments [21,23,24].

On another dimension, molecular responses have been increasingly considered in environmental sciences because of their pivotal role in mediating gene – environment interactions and understanding of transgenerational effects resulting from exposure [26,28,29]. Regarding freshwater biota, the crustacean *Daphnia magna* is a model organism in ecotoxicology, being widely considered in regulatory frameworks and recognized as a model invertebrate for environmental omics research [30–32]. While the molecular mechanisms of tolerance to copper remain fairly unexplored in *Daphnia*, previous studies demonstrated the capacity of this metal to modulate gene expression and promote epigenetic changes in a manner related to the history of exposure, and that such alterations supported known and uncovered novel mechanisms of metal toxicity [10,33,34]. Furthermore, it has been suggested that metals can potentially influence the gene expression profiles of non-exposed offspring, though this has never been experimentally confirmed for daphnids [35–37]. Indeed, this is no simple task in *Daphnia* and many other organisms, as transgenerational effects can only be confirmed in the third non-stressed generation (F3) since the environmental exposure of pregnant females (F0 generation) accounts also for the exposure of the progeny (future F1) and germ line of the progeny (future F2) to the same stressor [38–40]. In parallel, it has been increasingly argued that transcriptional and epigenetic responses hold the potential to serve as biological markers of exposure and effects, while being particularly relevant for the incorporation of transgenerational heritability into risk assessment procedures [24,41–43]. However, more studies are urgently required to unveil the transgenerational effects occurring due to chemical exposures, and clarify the role and possible connection between different molecular mechanisms (e.g., epigenetic and transcriptional responses) in such processes [41,44,45].

Considering all the above, the main goal of this work was to assess the transgenerational effects occurring due to copper (Cu) exposure in *D. magna* at the transcriptional level, specifically evaluating the influence of past exposure histories in gene expression, using a real-time PCR (RT-PCR) approach through an array with 40 genes involved in detoxification and antioxidant activities, DNA damage repair, circadian clock functioning and epigenetic regulation. Under this general goal, we hypothesized that (i) Cu can induce transgenerational effects in gene expression patterns; (ii) the gene expression responses to Cu exposure of naïve and non-naïve daphnids concerning previous acclimation to Cu will differ; (iii) the history of exposure interferes with the transgenerational effects in gene expression patterns.

## Material e methods

### *Daphnia* culturing

Monoclonal cultures of *Daphnia magna* (clone Beak) were used herein, reared at a temperature of 20 ± 2°C and 16 h/8 h light/dark photoperiod that was provided by cool fluorescent white lights, and in ASTM hard water medium [46] enriched with vitamins and supplemented with an organic additive based on *Ascophyllum nodosum*. The culture medium was renewed three times a week and, simultaneously, organisms were fed with *Raphidocelis subcapitata* suspensions (ration: 3 × 10^5^ cells mL^−1^) that were cyclically cultured in Woods Hole MBL [47].

As detailed elsewhere [34], the EC20 for Cu − 0.021 mg/L (0.018–0.026 mg/L confidence interval) – was estimated based on a range finding test [48]. Then, parallel bulk cultures differing in the concentration of Cu in the media were established (nominal Cu concentrations dosed as CuSO_4_ ∙ 5 H_2_O): 0 mg/L (cultures producing naïve daphnids; Cu^−^) or 0.021 mg Cu/L (cultures producing non-naïve daphnids; Cu^+^). These parallel cultures were reared as described above for three generations before initiating the multigenerational experiment (see Figure S1 for additional experimental details).

### Multigenerational experiment

Monoclonal cultures holding 70 neonates, ageing less than 24 h and collected from the 3^rd^ brood released in each parallel bulk culture, were established in plastic buckets holding 4 L of test solution (57 mL per organism) and incubated under the same conditions as described in section 2.1. Three experimental treatments were established in triplicate as the F0 generation: the control, composed of naïve organisms in blank ASTM medium (0 mg Cu/L); Cu^−/+^ composed of naïve daphnids in ASTM spiked at a Cu concentration of 0.021 mg/L; Cu^+/+^ composed of non-naïve daphnids in ASTM spiked at a Cu concentration of 0.021 mg/L (Figure S1).

Independently from the treatment held at the F0, the three following generations – F1 (coded as Cu^+/+^ or Cu^−/+^), F2 (coded as Cu^+/+/-^ or Cu^−/+/-^) and F3 (coded as Cu^+/+/-/-^ or Cu^−/+/-/-^) – consisted of organisms reared in blank ASTM, i.e., 0 mg/L of Cu (Figure S1). Each generation was defined as the period from an organism’s birth to the release of their 3^rd^ brood neonates, thus the neonates released from F0 were utilized for starting the F1 treatments; the same applying to the establishment of F2 from F1 and F3 from F2. Throughout the experiment, *Daphnia* mothers from F0 and F3 were pooled per replicate bucket shortly after releasing their 3^rd^ brood (empty brood pouches), and stored at − 80°C with RNAlater® for subsequent RNA extraction; this corresponds to the most cost-effective strategy to detect the true transgenerational inheritance of effects resulting from environmental exposures in *Daphnia* and many other organisms [39,40].

### RNA isolation and complementary DNA (cDNA) synthesis

Total RNA was extracted from batches of RNAlater®-preserved organisms (10 daphnids per condition and replicate) by employing the TRIzol™ reagent (Invitrogen™), as an improvement of the single-step RNA isolation method from Chomczynski and Sacchi [49], according to the manufacturer’s instructions. The isolated RNA was used to synthesize cDNA by retro-transcription using the Moloney Murine Leukemia Virus Reverse Transcriptase (M-MLV-RT) enzyme (Invitrogen^TM^) and the cDNA was stored at −20°C until further use. Protocol details for RNA isolation and cDNA synthesis are provided in Supplementary Section S1.

### Gene expression analysis

#### Array

An array was designed to analyse the transcriptional changes of 40 genes, and five endogenous references – primer sequences and their efficiencies can be found in the Supplementary Table S1, and efficiencies were calculated according to Ozáez et al. [50]. The set of 40 genes was rationally selected considering their established/putative involvement in the biological functions affected by Cu toxicity or mechanisms underpinning detoxification pathways and stress responses of invertebrates facing metal contamination [10,33,51]. The selection and its rationale are detailed in Supplementary Section S2.

#### RT-PCR protocol

The reaction was performed in a final volume of 10 μL, employing the cDNA as template for RT-PCR, with 0.5 units of DNA polymerase (Biotools, Spain), 0.4 mM of dNTPs (Biotools, Spain) and 0.5× Eva Green (Biotium, USA), in a CFX96 thermocycler (Bio-Rad, USA). The thermal cycling program consisted of an initial denaturation (95°C) for 5 s, followed by 40 cycles at 95°C for 15 s, 58°C annealing for 15 s, and 72°C elongation for 30 s. It included a melting curve analysis performed from 60 to 90°C with increment of 0.5°C to verify that only one band constituted each signal. Each sample was run in duplicate wells as technical replicates, and then each sample was run in two different microplates. Total messenger RNA (mRNA) levels of normalized gene expression were analysed by applying the (2^−ΔCq^) correction by relative quantification [52].

#### Statistical analysis

Statistical analysis was conducted in the software SPSS (IBM, USA), version 25. Normality and homogeneity of variances were assessed by performing, respectively, Shapiro-Wilk and Levene tests. Deviations from normality determined the usage of the non-parametric Kruskal-Wallis test followed by the post-hoc Mann-Whitney U. In all the cases, significant differences were fixed at *p* < 0.05 (full statistical summaries were reported in Supplementary Table S2).

## Results

### Overall gene expression effects

All the 40 genes analysed presented differential gene expression in more than one treatment comparison (Table S2). Specifically, only two genes were found in the comparison between Cu^+/+^ and Cu^−/+^ and three in the comparison between Cu^+/+^ and control (see Table S3). On the other hand, 13 genes were differentially expressed between Cu^−/+^ and control, with upregulation under the Cu^−/+^ treatment being observed for all (Table S3). Furthermore, the comparison of the Cu^+/+/-/-^ and Cu^−/+/-/-^ treatments with the control revealed a much higher modulation on gene expression than in their corresponding F0 treatments, with 27 genes being differentially expressed in each of these F3 treatments and 15 shared between them (Table S3). However, these 15 overlapped genes mostly showed contrasting gene expression patterns: upregulation in Cu^+/+/-/-^ and downregulation in Cu^−/+/-/-^ (both in comparison to the control; Table S3). Moreover, the direct comparison of F3 non-naïve and naïve treatments (Cu^+/+/-/-^ and Cu^−/±/-/-^) showed that 36 genes were differentially expressed, with almost all showing lower expression levels in the Cu^−/±/-/-^ treatment (Table S3).

Moreover, the comparison of F0 treatments with their corresponding F3 showed remarkable transgenerational differences between naïve and non-naïve organisms: non-naïve generations only presented four genes differentially expressed while naïve generations presented 39 (F0 vs. F3), with three genes overlapping between them (Table S3). Also important, the 39 genes detected in the comparison of naïve F0 and F3 presented lower expression levels in F3 (Cu^−/+/-/-^); three out of the four genes identified in the comparison of non-naïve F0 and F3 were comparatively overexpressed in F3 (Cu^+/+/-/-^; Table S3).

### Group-specific gene expression effects

#### DNA damage and repair

**Figure 1.**
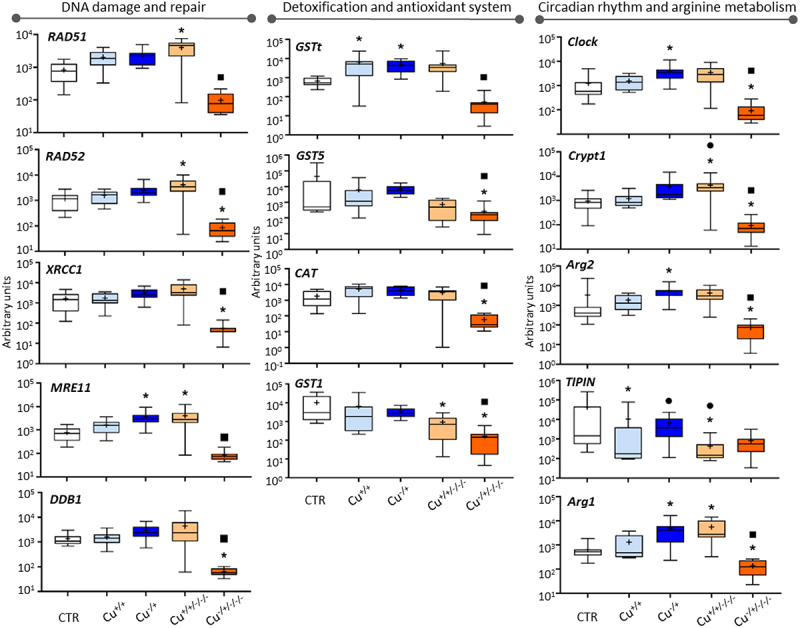
Box whisker plots representing mRNA levels regarding DNA damage and repair genes (left-hand panel), detoxification and antioxidant genes (central panel) and circadian rhythm and arginine metabolism genes (right-hand panel) in the experimental treatments. The horizontal line within the box indicates the median (plus sign locates the mean), the boundaries of the box the 25th and 75th percentiles, and the whiskers the highest and lowest results. Asterisks (*), circles (●) and squares (■) denote significant statistical differences (kruskal-Wallis; p-value <0.05) in gene expression in respect to the control, F0 non-naïve (Cu^+/+^) and naïve (Cu^−/+^) treatments, respectively.

The five genes involved in the recognition and repair of DNA damage showed consistent responses (Figure 1 – left-hand panel). First, there were no significant changes among treatments in F0 except for *MRE11*, which was found to be upregulated in the Cu^−/+^ in comparison to control. Second, DNA damage and repair genes were found typically upregulated in non-naïve F3 (Cu^+/+/-/-^) compared to control (see *RAD51*, *RAD52* and *MER11*), while downregulation was rather generally found for the naïve F3 (Cu^−/+/-/-^) as evident for *DDB1*, *RAD52* and *XRCC1*. Third, significantly lower expression levels were observed in the naïve F3 (Cu^−/+/-/-^) in comparison to the corresponding F0 generation, while non-naïve organisms retained expression levels from F0 to F3.

#### Detoxification and antioxidant system

The four genes with recognized roles in detoxification and antioxidant activity were not particularly responsive at F0 – only *GSTt* was upregulated compared to control in both non-naïve and naïve organisms (see Cu^+/+^ and Cu^−/+^ in Figure 1 – central panel). Concerning the F3 responsivity, *GST1* was differentially expressed relative to the control in non-naïve organisms (Cu^+/+/-/-^), while *CAT*, *GST1*, *GST5* were downregulated in naïve organisms (Cu^−/+/-/-^). Consistently to the transcriptional responses observed for most genes, transgenerational changes were observed for these four genes in naïve organisms (significantly lower expression always observed in F3 compared to F0), while genes kept expression levels transgenerationally in non-naïve organisms.

#### Circadian rhythm and arginine metabolism

Regarding the genes involved in the regulation of the circadian clock and arginine metabolism at the F0, *TIPIN* was differentially expressed both comparing Cu^+/+^ with the control, and Cu^+/+^ with Cu^−/+^, the Cu^+/+^ treatment always leading to lower expression (Figure 1 – right-hand panel); the expression of *Clock* and two Arginase genes was rather induced in the Cu^−/+^ treatment relative to the control. At F3, *Arg1*, *TIPIN* and *Crypt1* were differentially expressed compared to the control in non-naïve organisms (Cu^+/+/-/-^), while *Clock*, *Crypt1, Arg1* and *Arg2* were consistently downregulated in naïve organisms (Cu^−/+/-/-^). Transgenerational gene expression patterns (F0 vs. F3) differed between naïve and non-naïve organisms: two genes (*TINPIN* and *Crypt1*) were differentially expressed in non-naïve organisms; four out of five analysed were significantly less expressed in the naïve F3.

#### DNA methylation and non-coding RNAs

**Figure 2.**
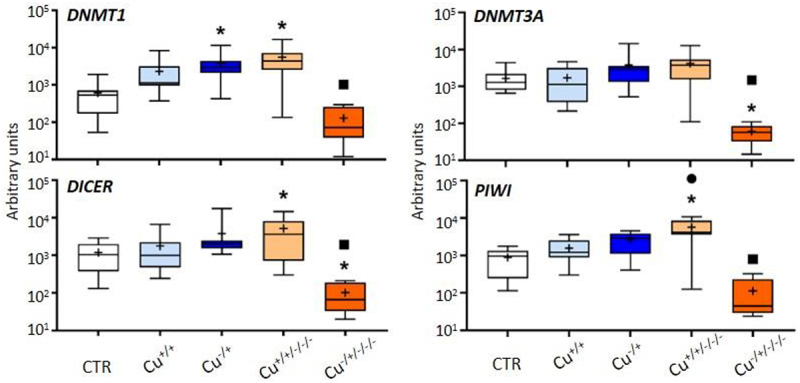
Box whisker plots representing mRNA levels of DNA methylation and non-coding RNAs genes in the experimental treatments. The horizontal line within the box indicates the median (plus sign locates the mean), the boundaries of the box the 25th and 75th percentiles, and the whiskers the highest and lowest results. Asterisks (*), circles (●) and squares (■) denote significant statistical differences (kruskal-Wallis; p-value <0.05) in gene expression in respect to the control, F0 non-naïve (Cu^+/+^) and naïve (Cu^−/+^) treatments, respectively.

The expression of genes involved in epigenetic regulation through DNA methylation and non-coding RNAs was unchanged among treatments at F0, with the exception of *DNMT1* that was found to be upregulated in naïve organisms (Cu^−/+^) in comparison to the control (Figure 2). At F3, *DNMT1*, *DICER* and *PIWI* were upregulated by the previous exposure to Cu in non-naïve daphnids, while *DICER* but then also *DNMT3A* were downregulated compared to the control in naïve daphnids. Once again, the transgenerational patterns of gene expression were distinct between naïve and non-naïve organisms. In these latter, only *PIWI* was found to be differentially expressed between F0 (Cu^+/+^) and F3 (Cu^+/+/-/-^), but for naïve organisms, the four genes assessed were significantly less expressed in the F3 (Cu^−/±/-/-^) compared to F0 (Cu^−/+^).

#### Histone modifications

**Figure 3.**
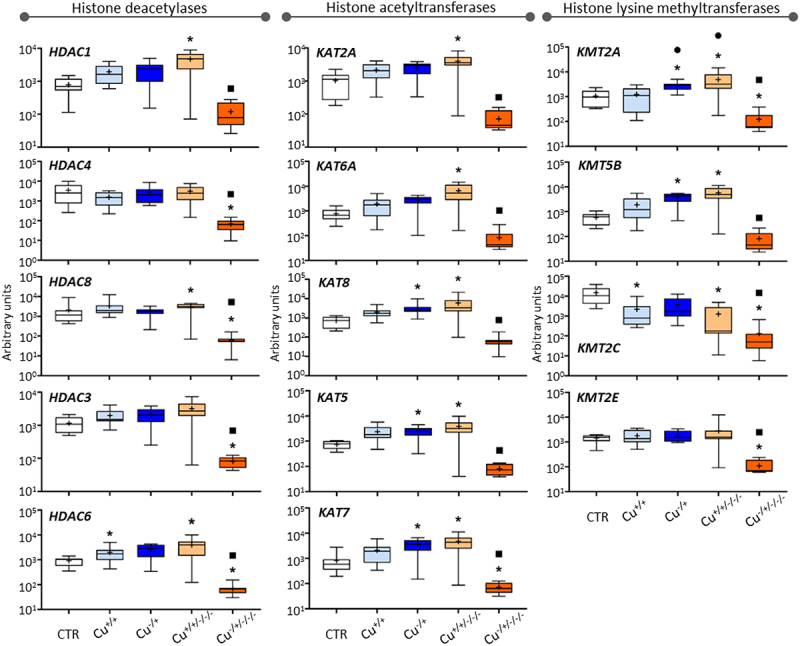
Box whisker plots representing mRNA levels concerning histone deacetylase genes (left-hand panel), histone acetyltransferase genes (central panel), and lysine methyltransferase genes (right-hand panel) in the experimental treatments. The horizontal line within the box indicates the median (plus sign locates the mean), the boundaries of the box the 25th and 75th percentiles, and the whiskers the highest and lowest results. Asterisks (*), circles (●) and squares (■) denote significant statistical differences (kruskal-Wallis; p-value <0.05) in gene expression in respect to the control, F0 non-naïve (Cu^+/+^) and naïve (Cu^−/+^) treatments, respectively.

The Histone deacetylase genes were not differentially expressed at F0 regardless of the comparison in place (Figure 3 – left-hand panel). However, *HDAC1, HDC6* and *HDC8* were upregulated in non-naïve daphnids at F3 (Cu^+/+/-/-^) compared to the control, while in naïve F3 daphnids (Cu^−/+/-/-^) downregulation was consistently found for all genes, except *HDAC1*. No gene was found differentially expressed between F3 and the corresponding F0 in non-naïve organisms, but all genes were significantly less expressed in the naïve F3 (Cu^−/±/-/-^) compared to the F0 (Cu^−/+^).

Histone acetyltransferase genes were unresponsive to Cu exposure in the non-naïve F0, while *KAT5*, *KAT7* and *KAT8* were upregulated in the naïve F0 (Cu^−/+^; Figure 3 – central panel). This pattern was inverted in the naïve F3 (Cu^−/±/-/-^), where these and the other assessed histone acetyltransferase genes were less expressed than the control and corresponding F0 (Cu^−/+^). All genes were upregulated in the non-naïve F3 (Cu^+/+/-/-^) compared to the control but none was differentially expressed compared to the corresponding F0, i.e., Cu^+/+^.

The four Lysine methyltransferase genes did not present the same transcriptional patterns as previously reported for other genes. Copper induced transcriptional repression of *KMT2C* in the non-naïve F0 (Cu^+/+^ vs. Control), while the genes *KMT2A* and *KMT5B* were upregulated in the naïve F0 (Cu^−/+^ vs. Control; Figure 3 – right-hand panel). At the F3 level, *KMT2A*, *KMT2C* and *KMT5B* were upregulated in non-naïve organisms (Cu^+/+/-/-^ vs. Control), and the expression of *KMT2A* significantly increased transgenerationally, i.e., comparing to the Cu^+/+^ treatment. Besides, all genes showed significantly lower expression in the Cu^−/±/-/-^ treatment in comparison to Cu^−/+^.

**Figure 4.**
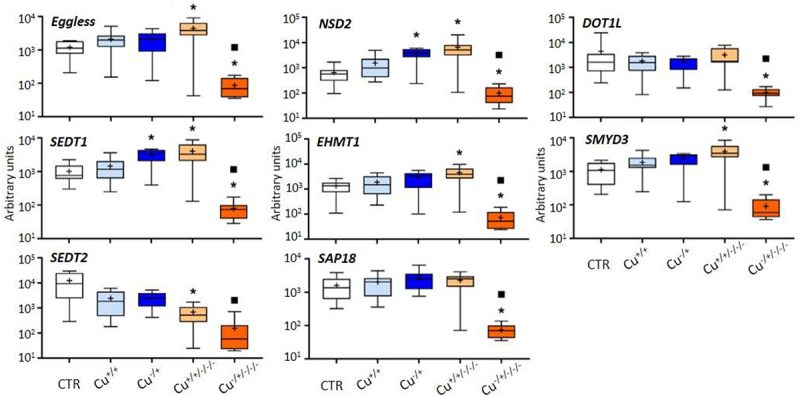
Box whisker plots representing mRNA levels of other histone modifier genes in the experimental treatments. The horizontal line within the box indicates the median (plus sign locates the mean), the boundaries of the box the 25th and 75th percentiles, and the whiskers the highest and lowest results. Asterisks (*) and squares (■) denote significant statistical differences (kruskal-Wallis; p-value <0.05) in gene expression in respect to the control, and F0 naïve (Cu^−/+^) treatments, respectively.

The other histone modifiers were consistently unresponsive at F0, similar to what was observed in general in this work; only *NSD2* and *SEDT1* were found to be upregulated in naïve daphnids (Cu^−/+^) compared to the control (Figure 4). On the other hand, five of the assessed genes were upregulated in the Cu^+/+/-/-^ treatment in comparison with the control, and all were downregulated in the Cu^−/±/-/-^ treatment. Again, naïve daphnids were transcriptionally more responsive at the F3 comparing to the corresponding F0, with all genes of this group showing significantly lower expression levels at F3 than at the corresponding F0 generation, i.e., Cu^−/+^.

## Discussion

The molecular mechanisms underpinning tolerance to Cu remain poorly understood for *Daphnia* and other invertebrates, and almost unexplored from a transgenerational perspective [10,33,37]. Nevertheless, previous studies focusing on *Daphnia* revealed that metal exposure induced gene expression modulation and epigenetic changes in a very small number genes, with toxicant-specific mRNA expression patterns being consistently reported [34,53,54]. Regardless of the history of exposure, we also found a mild immediate transcriptional response at the F0, with a low number of genes being differentially expressed compared to the control. However, much higher transcriptional responsivity was observed at F3, which shows that Cu exposure is rather able to affect daphnids transgenerationally, i.e., that the environmental susceptibility of truly non-exposed generations (F3) is a likely phenomenon extending Cu effects beyond directly exposed generations (F0, F1 and F2), thus confirming previous claims that long-lasting effects can occur at the transcriptional level following exposure to different metals, including essential metals [37,43,55].

In this regard, only three genes were found differentially expressed at the F0 of non-naïve daphnids (Cu^+/+^) as compared with expression levels in the Control: downregulation of a circadian rhythm and a histone modifier gene, and upregulation of an antioxidant gene. By contrast, a much higher number of genes was found differentially expressed at the F0 of the naïve organisms (Cu^−/+^), all upregulated compared to the Control, namely several involved in the circadian rhythm, arginine metabolism, DNA repair, epigenetic regulation (including a large number of histone modifier genes) and the same antioxidant gene identified in the comparison of Cu^+/+^ with Control. This general picture revealed that a single-generation exposure to Cu largely determined different immediate gene expression responses between non-naïve and naïve organisms, and that those organisms without an acclimation history to the stressor (naïve organisms), were more affected at the gene expression level than those previously acclimated (non-naïve ones). In agreement, the comparison between non-naïve and naïve F3 (respectively, Cu^+/+/-/-^ vs. Cu^−/+/-/-^) revealed that almost all genes were expressed at significantly lower levels in naïve organisms (Cu^−/+/-/-^), including a large number of genes involved in epigenetic regulation, circadian rhythm, DNA repair and antioxidant and detoxification mechanisms. Remarkably, this indicates that the different histories of exposure to Cu influenced the transcriptional responses and transgenerational extension of these responses to truly non-exposed generations (F3), thus showing that the molecular responses of newly exposed organisms may be critically constrained by their history of exposure to that same stressor [10,13].

### Stressor-specific responses: detoxification pathways

Detoxification mechanisms have been identified following the exposure of *Daphnia* and other invertebrates to metals, with these being related to their capacity to induce DNA damage and inhibit repair mechanisms, as well as to promote oxidative stress, which can provoke further DNA damage, protein degradation, lipid peroxidation or even cell damage and apoptosis through the action of reactive oxygen species [56–58]. The *GSTt* gene was upregulated at F0 following exposure to Cu of naïve and non-naïve organisms. The genes belonging to the Glutathione-S-transferase family are known to have a key role in invertebrates’ stress response, through the conjugation of reduced glutathione with xenobiotics towards detoxification and protection of cells from oxidative damage. Related genes were already shown to be transcriptionally up-regulated following metal exposure in *Daphnia* [51,59].

Signalling the background for the request of detoxification mechanisms, several genes involved in DNA repair were found differentially expressed when comparing Cu^+/+^ and the Control (e.g., *MRE11*), and between naïve and non-naïve F3 generations (e.g., *RAD52* and *RAD51*), thus reinforcing the capacity of Cu to directly induce DNA breaks or indirectly cause genomic instability through the formation of reactive oxidative species [60–62]. Supporting this rationale, previous studies demonstrated the overexpression of genes involved in DNA repair following the exposure of daphnids to metal-based chemicals or in the presence of enhanced oxidative stress [33,45,63]. Besides, transcriptional changes have been previously recorded for genes involved in immune responses and metabolism following *Daphnia* exposure to Cu [10,51]. This suggests that the transcriptional changes observed in both F0 and F3 comparisons for the *Arg1* and *Arg2* genes, which play a role in bioenergetic pathways and are considered potential regulators of immune responses in *Daphnia*, are involved in an increase of immune functions or the metabolic changes required to provide the cells energy to mitigate toxic effects [10,64,65]. Finally, transcriptional changes were induced by Cu in naïve and non-naïve F0 and F3 organisms for genes involved in the regulation of the circadian rhythm, which provides a source of internal timing in most living organisms [66,67]. Interestingly, the circadian rhythm is known to be a regulator of the oxidation – antioxidant balance in daphnids and the suppression of this system can serve as a trade-off for their adaptation to environmental stressors, thereby suggesting a possible connection between the observed changes for circadian rhythm and antioxidant genes [68,69].

### Evidenced links between epigenetic mechanisms and detoxification pathways

Epigenetic mechanisms are important actors in the detoxification and adaptation of *Daphnia* following exposure to chemicals, namely due to their contribution to fine-tune gene expression patterns under such scenarios [16,70]. Specifically, DNA methylation, which is the most well-studied epigenetic mechanism, was previously shown to be affected by Cu exposure in *Daphnia*, with methylation changes occurring in genes important for counteracting the toxic effects of metals and oxidative stress [34]. Herein, we observed transcriptional changes in *DNMT1* in the naïve F0 as a direct exposure effect, and then between F3 treatments (Cu^+/+/-/-^ vs. Cu^−/+/-/-^), this gene encoding an enzyme that is responsible for maintaining methylation patterns during DNA replication [71]. Besides, the *DICER* gene was found to be differentially expressed between naïve and non-naïve F3 organisms, and this codes for a protein that acts in the maturation of several non-coding RNAs that constitute another main epigenetic mechanism [72]. Remarkably, different classes of non-coding RNAs have been identified in *Daphnia* and their role in the response to chemicals experimentally demonstrated [73,74].

More evidence includes the detection of transcriptional changes for a great number of genes involved in the other major epigenetic mechanism, i.e., histone modifications, at F0 and F3 and for both non-naïve and naïve organisms. Some examples include differential expression of two Lysine methyltransferase (*KMT2A* and *KMT2C*) and several Lysine acetyltransferase genes (*KAT5*, *KAT7* and *KAT8*) at F0, these encoding proteins that catalyse the transfer of methyl and acetyl groups to histones, respectively [75,76]. Accordingly, different types of histone post-translational modifications (PTM) are known, and these can either promote or repress transcription according to the type and location of the modification, mainly by influencing the accessibility of the RNA polymerase to the promoter region of genes [77,78]. Besides, histone PTM are known to be determined by environmental stressors, and these can underpin aberrant transcriptional patterns that drive negative phenotypic effects; yet, there is also evidence that such modifications can sometimes serve as important regulators of stress responses in invertebrates, thus being crucial mechanisms to offset toxic effects [45,79,80]. Moreover, histone modifications can associate to DNA methylation changes to regulate gene expression in *Daphnia* in a very specific manner, while previous studies already showed the occurrence of histone modifications and transcriptional changes in histone PTM genes following enhanced oxidative stress in daphnids [29,35,45]. Taken together, these results showed the high likelihood of histone modifications resulting from *Daphnia* exposure to Cu, and reinforced previous evidence on the importance and possible interplay of different epigenetic mechanisms in the detoxification responses of invertebrates facing metal contamination [29,34,45].

### Transgenerational effects of Cu – the interference of exposure history

A remarkably distinct transcriptional pattern from F0 to F3 was noticed between non-naïve and naïve organisms. Specifically, 39 genes showed lower expression at the naïve F3 (in comparison to the corresponding F0 treatment), thus suggesting a general recovery response with the goal of restoring normal physiological levels and ceasing unnecessary energy spending in biological functions initially involved in coping with the stress insult at F0 [81,82]. In contrast, non-naïve daphnids largely maintained expression levels from F0 to F3, therefore indicating that their acclimation to Cu was complete at F0, and driven by their culturing in a Cu-enriched medium for several generations prior to the experiment.

Still, transgenerational differences in non-naïve organisms were found for four genes, including the overexpression of three of these at the F3 (Cu^+/+/-/-^ vs. Cu^+/+^): two (*TIPIN* and *Crypt 1*) involved in the regulation of the circadian rhythm and the other two (*PIWI* and *KMT2A*) playing a role in the action of non-coding RNAs and histone modifications [73,75,83]. Interestingly, these genes presented contrasting transgenerational responses between naïve F0 and F3 generations (Cu^−/+^ and Cu^−/±/-/-^), since there was no transcriptional change in *TIPIN*, while *Crypt 1, PIWI*, *KMT2A* showed decreased expression at the F3. The unique transgenerational gene expression responses in non-naïve daphnids were likely determined by their extended history of exposure (acclimation plus F0 exposure to Cu), potentially underpinning less demanding modulation of gene expression in future generations because such modulation already happened during acclimation, rendering them enhanced capacity to respond to a future exposure to the same stressor [10,34]. By contrast, due to their limited history of exposure to Cu, naïve organisms seemed to lack the capacity to invest in the retention of a transgenerational memory from the stressor exposure [34].

Interestingly, numerous studies demonstrated that the functioning of the circadian rhythm relies upon transcriptional loops that are regulated by epigenetic mechanisms [84,85]. Besides, environmental cues can alter circadian rhythms, which may lead to the initiation of several diseases, though cells can experience epigenetic alterations to fine-tune their transcriptional rhythms in accordance to the external environment [84,86]. Such evidence further supports the view that the mentioned transgenerational transcriptional responses for genes involved in epigenetic regulation and circadian clock may constitute a stressor-adaptation response in organisms acclimated in Cu, aimed at allowing the non-exposed generations better equipped to cope with a future exposure to the same stressor [34,86]. More compelling evidence includes the detection of DNA methylation inheritance and unique life-history responses from non-exposed generations when *Daphnia* was exposed to Cu in a similar experimental setup, i.e., organisms acclimated in a Cu-enriched medium vs. blank ASTM [34]. Similarly to the transgenerational transcriptional changes detected here, such methylation inheritance occurred in a manner related explicitly to the history of exposure, thus being different for non-naïve and naïve generations, and mainly targeted genes that offset metal and oxidative stress toxicity [34]. Interestingly, the cross-talk between the antioxidative system and circadian clocks through epigenetic regulation of gene expression is already known, thus making possible that a connection exists between the transgenerational DNA methylation and transcriptional responses above reported, especially because gene expression in invertebrates is under the direct control of epigenetic mechanisms [34,35,86]. In this regard, the future analysis of biochemical and other organismal higher-level effects would be important to better understand the functional consequences of the gene expression changes reported in this work and their connection to the related epigenetic responses described in the literature.

**Figure 5.**
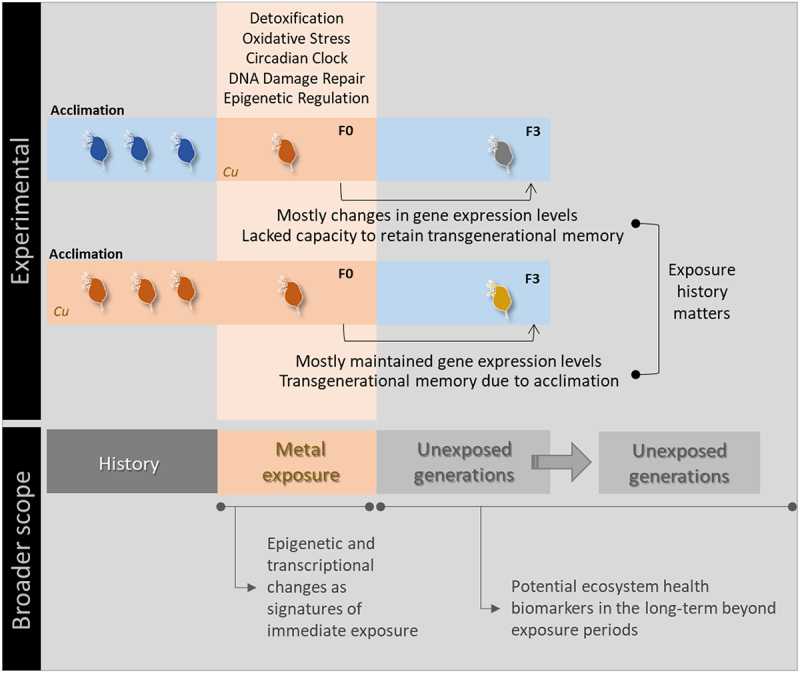
Schematic representation of the main findings of the study and their relation to the broader environmental assessment context.

Contrasting transcriptional responses to copper were found between non-naïve and naïve organisms at the F0 and F3 generations, with remarkably different transgenerational gene expression responses being also observed between both groups (Figure 5). These results brought into light the effects of past exposure to stressors on gene expression, and revealed that metals can greatly influence the transcriptional responses of truly non-exposed generations, thereby opening new perspectives for the environmental assessment and monitoring of different metals, especially over the long term (Figure 5). Additionally, and regardless of treatments focused, histone modifier genes were always found transcriptionally altered, thus suggesting that epigenetic regulation plays a central role in the transgenerational and stress responses of *Daphnia* facing metal exposure. Overall, this evidence makes the case for the future understanding of epigenetic and transcriptional changes as signatures of exposure, and their incorporation (in combination to transgenerational inheritance) into risk assessment procedures.

## Supplementary Material

-)Revised_SupplementaryMaterial_Jeremias2023_Final.doc

## Data Availability

The authors confirm that the data supporting the findings of this study are available within the article and its supplementary materials.

## Author CRediT roles

GJ: Conceptualization, Investigation, Methodology, Writing – original draft. A-B M-G: Formal analysis, Investigation, Methodology, Writing – review & editing. FJMG: Supervision, Writing – review & editing. J-L M-G: Funding acquisition, Writing – review & editing. JA: Conceptualization, Funding acquisition, Supervision, Writing – review & editing. JLP: Conceptualization, Funding acquisition, Supervision, Writing – review & editing.
