# Supplementary material for: History of exposure to copper influences transgenerational gene expression responses in Daphnia magna
Source: Epigenetics. 2023 Dec 28;19(1):2296275. doi: 10.1080/15592294.2023.2296275 (PMC10761054; doi:10.1080/15592294.2023.2296275)
Supplement: -)Revised_SupplementaryMaterial_Jeremias2023_Final.doc [file KEPI_A_2296275_SM9792.doc]

**SUPPLEMENTARY MATERIAL**

**History of exposure to copper influences transgenerational gene expression responses in *Daphnia magna***

Guilherme Jeremias (jeremias@ua.pt), Ana-Belén Muñiz-González (anabmglez@ccia.uned.es), Fernando José Mendes Gonçalves, José-Luis Martínez-Guitarte, Jana Asselman and Joana Luísa Pereira

**Section S1. Detailed protocol on RNA isolation and complementary DNA (cDNA) synthesis**

Firstly, the samples were thawed on ice and then the RNAlater® was removed. Secondly, the organisms were frozen again and then homogenised in 400 μL of TRIzol. After that, 80 µL of chloroform was added to the homogenate, the suspension was thoroughly mixed, incubated for 2-3 min at room temperature and centrifuged at 10,000 rpm at 4 °C. The aqueous upper phase was recovered, RNA was precipitated with isopropanol and washed with 70 % ethanol. The resultant pellet was treated with RNAse-free DNAase I (Roche, Germany) to eliminate any DNA traces, followed by a phenol/chloroform/isoamyl extraction with Phase Lock Light tubes (Quantabio, USA). The isolated RNA was resuspended in 25 μL of diethyl pyrocarbonate (DEPC)-treated water, quantified by UV spectrometry (Biphotometer, Eppendorf) and stored at −80 °C. Thereafter, RNA was used to synthesize cDNA by retro-transcription using the Moloney Murine Leukemia Virus Reverse Transcriptase (M-MLV-RT) enzyme (Invitrogen^TM^). Reactions contained 5 μg of RNA, which served as a template, 100 units of M-MLV enzyme, 0.5 μg poly dT20 (Macrogen, Korea), and 0.5 mM dNTPs (Biotools, Spain), with a final volume of 40 μL. Firstly, the reaction was activated at 37°C for 50 min and then it was stopped by incubating at 70 °C for 15 min. Finally, the cDNA was stored at -20 °C until further use.

**Section S2. Selection of genes for the gene expression array**

In particular, five selected genes were involved in the recognition and repair of DNA damage -DNA repair protein RAD52 homolog (*RAD52*), DNA repair protein RAD51 homolog (*RAD51*), Damage specific DNA binding protein 1 (*DDB1*), MRE11 homolog, double strand break repair nuclease, meiotic recombination (*MRE11*) and X-ray repair cross complementing 1 (*XRCC1*) (Asselman et al., 2019; Sekelsky, 2017). Four genes were selected as per its role in detoxification and antioxidant systems, namely the Catalase (*CAT*) and three Glutathione S-transferase family genes, including the Glutathion-S-transferase theta (*GSTt*), Glutathion-S-transferase theta 1 (*GST1*) and Glutathion-S-transferase theta 5 (*GST5*) (Asselman et al., 2019, 2013; Chain et al., 2019). Besides, three genes were selected because of their pivotal roles in the regulation and function of the circadian clock - Clock circadian regulator (*Clock*), TIMELESS interacting protein (*TIPIN*), and Cryptochrome 1 (*Crypt1*) -, as well as 2 genes involved in the arginine metabolism, namely the Arginase 1 (*Arg1*) and Arginase 2 (*Arg2*) (Cai et al., 2020; Decaestecker et al., 2011). Finally, the set included genes involved in epigenetic regulation because such molecular mechanisms are known to play a critical role in the detoxification and adaptation of *Daphnia* to different chemical exposures (Hearn et al., 2018; Jeremias et al., 2018; Thaulow et al., 2020), while it has been previously established that daphnids differing in their history of exposure to Cu presented contrasting DNA methylation transgenerational responses (Jeremias et al., 2022). Accordingly, regarding DNA methylation and non-coding RNAs, DNA methyltransferase 1 (*DNMT1*) and DNA methyltransferase 3 alpha (*DNMT3A*), Protein piwi (*PIWI*) and Dicer 1, ribonuclease III (*DICER*) were included (Foulkes et al., 2014; Schermelleh et al., 2007). A large number of genes involved in histone modifications was considered, including five Histone deacetylases (*HDAC1*, *HDAC3*, *HDAC4*, *HDAC6* and *HDAC8*), five Histone acetyltransferases (*KAT2A*, *KAT5*, *KAT6A*, *KAT7* and *KAT8*) and four Lysine methyltransferase genes (*KMT2A*, *KMT2C*, *KMT2E* and *KMT5B*) [61–63]. Histone methyltransferase activity (*Eggless)*, Euchromatic histone lysine methyltransferase 1 (*EHMT1*), DOT1 like histone lysine methyltransferase (*DOT1L*), Nuclear receptor binding SET domain protein 2 (*NSD2*), SET domain containing 1A, histone lysine methyltransferase (*SEDT1*) and SET domain containing 2, histone lysine methyltransferase (*SEDT2*), SET and MYND domain containing 3 (*SMYD3*) and Histone deacetylase complex subunit SAP18 (*SAP18*) were added considering their role as histone modifiers (Bannister and Kouzarides, 2011; Lawrence et al., 2016).

**References**

Asselman, J., Semmouri, I., Jackson, C.E., Keith, N., Van Nieuwerburgh, F., Deforce, D., Shaw, J.R., De Schamphelaere, K.A.C., 2019. Genome-Wide Stress Responses to Copper and Arsenic in a Field Population of Daphnia. Environ. Sci. Technol. 53, 3850–3859. https://doi.org/10.1021/acs.est.8b06720

Asselman, J., Shaw, J.R., Glaholt, S.P., Colbourne, J.K., De Schamphelaere, K.A.C., 2013. Transcription patterns of genes encoding four metallothionein homologs in Daphnia pulex exposed to copper and cadmium are time- and homolog-dependent. Aquat. Toxicol. 142–143, 422–430. https://doi.org/10.1016/j.aquatox.2013.09.010

Bannister, A.J., Kouzarides, T., 2011. Regulation of chromatin by histone modifications. Cell Res. 21, 381–395. https://doi.org/10.1038/cr.2011.22

Cai, M., Liu, Z., Yu, P., Jiao, Y., Chen, Q., Jiang, Q., Zhao, Y., 2020. Circadian rhythm regulation of the oxidation–antioxidant balance in Daphnia pulex. Comp. Biochem. Physiol. Part - B Biochem. Mol. Biol. 240, 110387. https://doi.org/10.1016/j.cbpb.2019.110387

Chain, F.J.J., Finlayson, S., Crease, T., Cristescu, M., 2019. Variation in transcriptional responses to copper exposure across Daphnia pulex lineages. Aquat. Toxicol. 210, 85–97. https://doi.org/10.1016/j.aquatox.2019.02.016

Decaestecker, E., Labbé, P., Ellegaard, K., Allen, J.E., Little, T.J., 2011. Candidate innate immune system gene expression in the ecological model Daphnia. Dev. Comp. Immunol. 35, 1068–1077. https://doi.org/10.1016/j.dci.2011.04.004

Foulkes, W.D., Priest, J.R., Duchaine, T.F., 2014. DICER1: mutations, microRNAs and mechanisms. Nat. Rev. Cancer 14, 662–672. https://doi.org/10.1038/nrc3802

Hearn, J., Chow, F.W.N., Barton, H., Tung, M., Wilson, P.J., Blaxter, M., Buck, A., Little, T.J., 2018. Daphnia magna microRNAs respond to nutritional stress and ageing but are not transgenerational. Mol. Ecol. 27, 1402–1412. https://doi.org/10.1111/mec.14525

Jeremias, G., Barbosa, J., Marques, S.M., De Schamphelaere, K.A.C., Van Nieuwerburgh, F., Deforce, D., Gonçalves, F.J.M., Pereira, J.L., Asselman, J., 2018. Transgenerational Inheritance of DNA Hypomethylation in Daphnia magna in Response to Salinity Stress. Environ. Sci. Technol. 52, 10114–10123. https://doi.org/10.1021/acs.est.8b03225

Jeremias, G., Veloso, T., Gonçalves, F.J.M., Nieuwerburgh, F. Van, Luísa, J., Asselman, J., 2022. Multigenerational DNA methylation responses to copper exposure in Daphnia: Potential targets for epigenetic biomarkers? Chemosphere 308, 136231. https://doi.org/10.1016/j.chemosphere.2022.136231

Lawrence, M., Daujat, S., Schneider, R., 2016. Lateral Thinking: How Histone Modifications Regulate Gene Expression. Trends Genet. 32, 42–56. https://doi.org/10.1016/j.tig.2015.10.007

Schermelleh, L., Haemmer, A., Spada, F., Rösing, N., Meilinger, D., Rothbauer, U., Cardoso, C.M., Leonhardt, H., 2007. Dynamics of Dnmt1 interaction with the replication machinery and its role in postreplicative maintenance of DNA methylation. Nucleic Acids Res. 35, 4301–4312. https://doi.org/10.1093/nar/gkm432

Sekelsky, J., 2017. DNA repair in Drosophila: Mutagens, models, and missing genes. Genetics 205, 471–490. https://doi.org/10.1534/genetics.116.186759

Thaulow, J., Song, Y., Lindeman, L.C., Kamstra, J.H., Lee, Y.K., Xie, L., Aleström, P., Salbu, B., Tollefsen, K.E., 2020. Epigenetic, transcriptional and phenotypic responses in Daphnia magna exposed to low-level ionizing radiation. Environ. Res. 190. https://doi.org/10.1016/j.envres.2020.109930


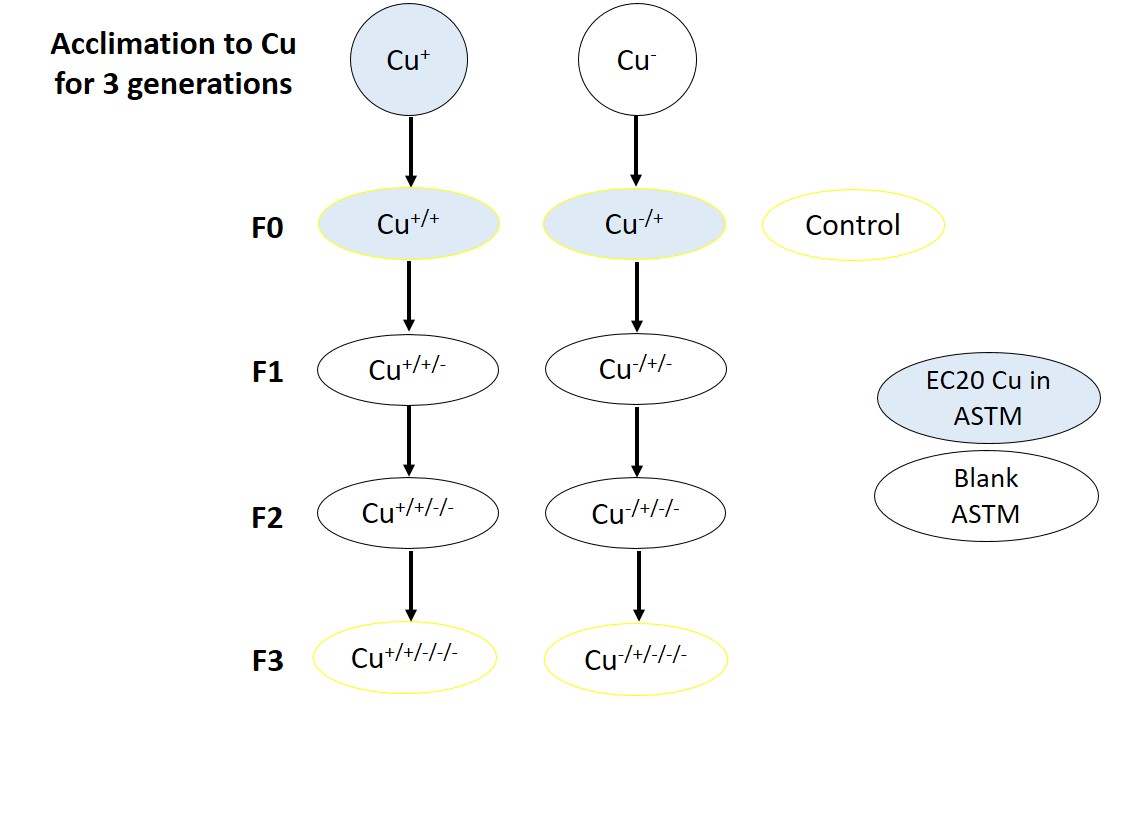


**Figure S1.** Multigenerational experimental design, comprising the acclimation of cultures in a Cu enriched medium or blank ASTM for three generations to simulate differential contamination history, the F0 exposure to the Cu enriched medium and the recovery (F1-F3) of organisms in blank ASTM. Respectively, white and blue filled boxes portray a clean medium (blank ASTM, 0 mg/L of Cu) and the EC20 for Cu (0.021 mg/L of Cu dissolved in ASTM using CuSO_4_ ∙ 5H_2_O). Yellow countered boxes indicate the treatments from which RNA was extracted and gene expression patterns assessed. Arrows stand for the start of new generations with 3^rd^ brood neonates from precedent cultures. Plus, and minus signs specify on the present and past culturing of organisms in a Cu enriched (+) or blank ASTM (−) medium.

**Table S1.** Primer sequence and PCR efficiency for the set of genes tested. The reference genes are highlighted in bold.

|  | **Gene** | **Forward** | **Reverse** | **Efficiency (%)** |
| --- | --- | --- | --- | --- |
| 1 | *Clock* | AGTCACTCACGAGCCAATCG | AGTTCCGTCTCAACACGTCG | 90,35 |
| 2 | *TIPIN* | ACCGTATTGCTGAAATGGTGG | GGAACTTACGGACTTCGCCA | 115,8 |
| 3 | *Crypt1* | TAGACGCTGACTGGTCGTTG | TTGCCAAAGCTATTGGGCT | 94,1 |
| 4 | *PIWI* | GCCGTTCCGTCTCAGATGAT | GCTAACAGCCCAAAGTTCGC | 99,3 |
| 5 | *Arg1* | CAATTGCCTGCTTTGCACCA | CATGCCCGCATCATTGCTTA | 105,7 |
| 6 | *Arg2* | ATCCCCGTGCCTCTCAGTAT | TCCTGTGGACGTTGTTCCAG | 97,3 |
| 7 | *DICER* | TTCTAGGCAATGCCGTGTTT | AGACGGCGAACGTGAACAATTA | 91,65 |
| 8 | *HDAC1* | TTGGACGTATGAGACTGCGG | TTCGACGGACTGATGTGCAG | 80 |
| 9 | *HDAC3* | CGGATGATTGAACACGCACC | GATGCAGGCGAGAATCAGGA | 86,55 |
| 10 | *HDAC4* | AACCAGTGCCCATTCGTCAA | ATGCGACCGAATAGGCGTAG | 83,3 |
| 11 | *HDAC6* | GAAGCCCCTCCTCCAAAGAC | TCATCATCGGGACGAACGTG | 106 |
| 12 | *HDAC8* | TTGCCCAGTCTGTACCTTGG | TTGGGCGTACCAATCGAAA | 93 |
| 13 | *KMT2A* | CTGGCTCCACTTTTGGTTGC | TGGCTGTTGAGCGTCTTGAT | 105,7 |
| 14 | *KMT2C* | TCCCACCGATTATCGCATCG | CCAACACTTGCTGACTTGGC | 83,8 |
| 15 | *KMT2E* | CGCCGACAAAAGAAGAACCG | CCTCGTTAACATGGCTGGGT | 89,4 |
| 16 | *Eggless* | CCGGGTCGAAAAACTACCCA | CTGACCATCTTCTTGGCCGT | 103,3 |
| 17 | *EHMT1* | TGCCAATGTCATCGAGGGTC | CTGACTCCCCAGCCTTTGTT | 96,25 |
| 18 | *DOT1L* | GAACCGGAACGTGAAGGACT | GGATAGATGGCGACGAAGGG | 95,65 |
| 19 | *NSD2* | CATTGGAGCCAAACCCAAGC | TCCGTACAGCGGAAGCAAAA | 76,7 |
| 20 | *SEDT1* | GACACGCATTGGGTGGACTA | GTGCCGATCCAGACTTGTGA | 100,6 |
| 21 | *SEDT2* | GATTTGGTTGCCATCGACCG | ATGGAAGACGTGGAAGAGCG | 96,6 |
| 22 | *SMYD3* | ATGAACGAACCGCATCCCTT | CGTTTCAACTCGCAGCAGTC | 94,5 |
| 23 | *KMT5B* | TCCTGTCCTGGACGAAGTCA | TGGGCTTGTTCCTGTTCGTT | 101,1 |
| 24 | *KAT2A* | GCAAGCATCGAACTTTGGCA | GCTCCGAGGATGTAACAGCA | 104,4 |
| 25 | *KAT5* | TCCAGTGAAGGTGAACGACC | GTACGTACTGCCCCTTGTAGT | 109,6 |
| 26 | *KAT6A* | CCTCCGTTCACGAGACCAAA | TGACGGAATCATCGCCACAA | 90,95 |
| 27 | *KAT7* | GGCTGTCACAGTGTCGGTTA | AGCAGTCGCCCATATCCTTG | 90,1 |
| 28 | *KAT8* | AGCCTCTTTCGGATTTGGGG | ATCGCTCTGCGTGATACTCG | 84 |
| 29 | *SAP18* | GAACTCCGCTCACCGTCTAC | CACGCAAATTTCCGCATGGA | 94,65 |
| 30 | *DNMT1* | TCCATATCGCACCGTCACTG | TCCAGCGCTGAAAATGGGAT | 90,7 |
| 31 | *DNMT3A* | TGACCAATCACGCGACCTAC | TGAATCCACAACGAGACGGG | 98,5 |
| 32 | *RAD52* | CCTTTGCCCTGGTTAACGGA | AGGGCGCTAATTTTGTTGTCG | 103,35 |
| 33 | *RAD51* | CAGCGCTCTTACAGGACTGAC | CGACTTGAGCGACTACCTGG | 92,35 |
| 34 | *DDB1* | CAAAGTTGAACCGTGCGGAGGG | CATTCCACTTCCGTCGTCCA | 96,5 |
| 35 | *MRE1* | TCAGGCTTTCGCTCGACAAT | TTAGGACGAGCAGAACGAGC | 89,4 |
| 36 | *XRCC1* | GCTACGCTAAGCGAAAACGG | TCGGATTCTGGTCCTCTGGT | 85,15 |
| 37 | *CAT* | TTTCTGTTCTCCGACCGTGG | TTGCCTCAATACCCGGAACC | 85,45 |
| 38 | *GST1* | TCACTGCCAAAATGGTGGGT | AAACAACCAATCAAGGGCGAC | 94,05 |
| 39 | *GST5* | TAATCCATGCTGGCGTGCTA | CCTTATCCCGGTGCTGGTAG | 85,3 |
| 40 | *GSTt* | GTCACAACCGTCTAGGGCG | GTTGCCTTTCGCACACGTTC | 81,7 |
| 41 | ***rpL11*** | GCTGGAACCATTTCATGGCA | TATGTTGTCCTTCGACGCCC | 81,55 |
| 42 | ***rpL09*** | GGTGTCTCAGACCGTCAAAGT | AGTGTTTTGAAATCGCGGGTC | 91,2 |
| 43 | ***TBP*** | CGTTCTGACTCACAGCCAGT | GAACTTTGGCGCCAGTAAGC | 79,3 |
| 44 | ***phfk*** | ATGAACGTTCTCGGCCACAT | TTGTGTGGCTTGGGTCAGTT | 90,8 |
| 45 | ***GAPDH*** | TCGTGGTGCTGCCCAAAATA | GAAGACAGGTCAGATCAACA | 88,35 |

**Table S2.** Significant gene-specific expression differences (p-value < 0.05) for the most relevant treatments. Comparison and Treatments illustrate the generations and specific treatments being compared, respectively. The test statistic, standard error and statistical deviance of the post-hoc Mann-Whitney U tests are provided.

| **Gene** | **Comparison** | **Treatment** | **Test Statistic** | **Standard Error** | **Statistical Deviance** | **P-value** |
| --- | --- | --- | --- | --- | --- | --- |
| Clock circadian regulator | F0 vs. F0 | Control vs. Cu^-/+^ | 12,750 | 5,990 | 2,129 | 0,033 |
|  | F0 vs. F3 | Control vs. Cu^-/+/-/-/-^ | 13,125 | 5,990 | 2,191 | 0,028 |
|  | F3 vs. F3 | Cu^+/+/-/-/-^ vs. Cu^-/+/-/-/-^ | 23,681 | 5,821 | 4,068 | 0,000 |
|  | F0 vs. F3 | Cu^-/+^ vs. Cu^-/+/-/-/-^ | 25,875 | 5,990 | 4,320 | 0,000 |
| TIMELESS interacting protein | F0 vs. F0 | Control vs. Cu^+/+^ | -12,250 | 5,990 | -2,045 | 0,041 |
|  | F0 vs. F0 | Cu^+/+^ vs. Cu^-/+^ | -13,375 | 5,990 | -2,233 | 0,026 |
|  | F0 vs. F3 | Control vs. Cu^+/+/-/-/-^ | 15,500 | 5,821 | 2,663 | 0,008 |
|  | F3 vs. F3 | Cu^+/+/-/-/-^ vs. Cu^-/+/-/-/-^ | -5,625 | 5,821 | -0,966 | 0,003 |
|  | F0 vs. F3 | Cu^+/+^ vs. Cu^+/+/-/-/-^ | 3,250 | 5,821 | 0,558 | 0,006 |
| Cryptochrome 1 | F0 vs. F3 | Control vs. Cu^+/+/-/-/-^ | -14,167 | 5,821 | -2,434 | 0,015 |
|  | F0 vs. F3 | Control vs. Cu^-/+/-/-/-^ | 12,125 | 5,990 | 2,024 | 0,043 |
|  | F3 vs. F3 | Cu^+/+/-/-/-^ vs. Cu^-/+/-/-/-^ | 26,292 | 5,821 | 4,517 | 0,000 |
|  | F0 vs. F3 | Cu^+/+^ vs. Cu^+/+/-/-/-^ | -11,792 | 5,821 | -2,026 | 0,043 |
|  | F0 vs. F3 | Cu^-/+^ vs. Cu^-/+/-/-/-^ | 23,875 | 5,990 | 3,986 | 0,000 |
| Protein piwi | F0 vs. F3 | Control vs. Cu^+/+/-/-/-^ | -16,847 | 5,821 | -2,894 | 0,004 |
|  | F3 vs. F3 | Cu^+/+/-/-/-^ vs. Cu^-/+/-/-/-^ | 27,472 | 5,821 | 4,720 | 0,000 |
|  | F0 vs. F3 | Cu^+/+^ vs. Cu^+/+/-/-/-^ | -11,972 | 5,821 | -2,057 | 0,040 |
|  | F0 vs. F3 | Cu^-/+^ vs. Cu^-/+/-/-/-^ | 21,125 | 5,990 | 3,527 | 0,000 |
| Arginase 1 | F0 vs. F0 | Control vs. Cu^-/+^ | 12,250 | 5,990 | 2,045 | 0,041 |
|  | F0 vs. F3 | Control vs. Cu^+/+/-/-/-^ | -12,444 | 5,821 | -2,138 | 0,033 |
|  | F0 vs. F3 | Control vs. Cu^-/+/-/-/-^ | 13,000 | 5,990 | 2,170 | 0,030 |
|  | F3 vs. F3 | Cu^+/+/-/-/-^ vs. Cu^-/+/-/-/-^ | 25,444 | 5,821 | 4,371 | 0,000 |
|  | F0 vs. F3 | Cu^-/+^ vs. Cu^-/+/-/-/-^ | 25,250 | 5,990 | 4,216 | 0,000 |
| Arginase 2 | F0 vs. F0 | Control vs. Cu^-/+^ | 14,625 | 5,990 | 2,442 | 0,015 |
|  | F0 vs. F3 | Control vs. Cu^-/+/-/-/-^ | 12,625 | 5,990 | 2,108 | 0,035 |
|  | F3 vs. F3 | Cu^+/+/-/-/-^ vs. Cu^-/+/-/-/-^ | 23,486 | 5,821 | 4,035 | 0,000 |
|  | F0 vs. F3 | Cu^-/+^ vs. Cu^-/+/-/-/-^ | 27,250 | 5,990 | 4,550 | 0,000 |
| Dicer 1, ribonuclease III | F0 vs. F3 | Control vs. Cu^+/+/-/-/-^ | -9,917 | 5,821 | -1,704 | 0,088 |
|  | F0 vs. F3 | Control vs. Cu^-/+/-/-/-^ | 14,875 | 5,990 | 2,483 | 0,013 |
|  | F3 vs. F3 | Cu^+/+/-/-/-^ vs. Cu^-/+/-/-/-^ | 24,792 | 5,821 | 4,259 | 0,000 |
|  | F0 vs. F3 | Cu^-/+^ vs. Cu^-/+/-/-/-^ | 23,625 | 5,990 | 3,944 | 0,000 |
| Histone deacetylase 1 | F0 vs. F3 | Control vs. Cu^+/+/-/-/-^ | -15,778 | 5,821 | -2,711 | 0,007 |
|  | F3 vs. F3 | Cu^+/+/-/-/-^ vs. Cu^-/+/-/-/-^ | 26,028 | 5,821 | 4,471 | 0,000 |
|  | F0 vs. F3 | Cu^-/+^ vs. Cu^-/+/-/-/-^ | 20,250 | 5,990 | 3,381 | 0,001 |
| Histone deacetylase 3 | F0 vs. F3 | Control vs. Cu^-/+/-/-/-^ | 12,250 | 5,990 | 2,045 | 0,041 |
|  | F3 vs. F3 | Cu^+/+/-/-/-^ vs. Cu^-/+/-/-/-^ | 25,319 | 5,821 | 4,350 | 0,000 |
|  | F0 vs. F3 | Cu^-/+^ vs. Cu^-/+/-/-/-^ | 20,875 | 5,990 | 3,485 | 0,000 |
| Histone deacetylase 4 | F0 vs. F3 | Control vs. Cu^-/+/-/-/-^ | 21,750 | 5,990 | 3,631 | 0,000 |
|  | F3 vs. F3 | Cu^+/+/-/-/-^ vs. Cu^-/+/-/-/-^ | 21,819 | 5,821 | 3,749 | 0,000 |
|  | F0 vs. F3 | Cu^-/+^ vs. Cu^-/+/-/-/-^ | 21,250 | 5,990 | 3,548 | 0,000 |
| Histone deacetylase 6 | F0 vs. F3 | Control vs. Cu^+/+/-/-/-^ | -13,806 | 5,821 | -2,372 | 0,018 |
|  | F0 vs. F3 | Control vs. Cu^-/+/-/-/-^ | 12,125 | 5,990 | 2,024 | 0,043 |
|  | F3 vs. F3 | Cu^+/+/-/-/-^ vs. Cu^-/+/-/-/-^ | 25,931 | 5,821 | 4,455 | 0,000 |
|  | F0 vs. F3 | Cu^-/+^ vs. Cu^-/+/-/-/-^ | 22,875 | 5,990 | 3,819 | 0,000 |
| Histone deacetylase 8 | F0 vs. F3 | Control vs. Cu^+/+/-/-/-^ | -11,861 | 5,821 | -2,038 | 0,042 |
|  | F0 vs. F3 | Control vs. Cu^-/+/-/-/-^ | 14,500 | 5,990 | 2,421 | 0,015 |
|  | F3 vs. F3 | Cu^+/+/-/-/-^ vs. Cu^-/+/-/-/-^ | 26,361 | 5,821 | 4,529 | 0,000 |
|  | F0 vs. F3 | Cu^-/+^ vs. Cu^-/+/-/-/-^ | 17,750 | 5,990 | 2,963 | 0,003 |
| Lysine methyltransferase 2A | F0 vs. F0 | Control vs. Cu^-/+^ | 12,000 | 5,990 | 2,003 | 0,045 |
|  | F0 vs. F0 | Cu^+/+^ vs. Cu^-/+^ | -11,875 | 5,990 | -1,983 | 0,047 |
|  | F0 vs. F3 | Control vs. Cu^+/+/-/-/-^ | -12,972 | 5,821 | -2,229 | 0,026 |
|  | F0 vs. F3 | Control vs. Cu^-/+/-/-/-^ | 12,625 | 5,990 | 2,108 | 0,035 |
|  | F3 vs. F3 | Cu^+/+/-/-/-^ vs. Cu^-/+/-/-/-^ | 25,597 | 5,821 | 4,398 | 0,000 |
|  | F0 vs. F3 | Cu^+/+^ vs. Cu^+/+/-/-/-^ | -12,847 | 5,821 | -2,207 | 0,027 |
|  | F0 vs. F3 | Cu^-/+^ vs. Cu^-/+/-/-/-^ | 24,625 | 5,990 | 4,111 | 0,000 |
| Lysine methyltransferase 2C | F0 vs. F0 | Control vs. Cu^+/+^ | -12,750 | 5,990 | -2,129 | 0,033 |
|  | F0 vs. F3 | Control vs. Cu^+/+/-/-/-^ | 17,722 | 5,821 | 3,045 | 0,002 |
|  | F0 vs. F3 | Control vs. Cu^-/+/-/-/-^ | 27,625 | 5,990 | 4,612 | 0,000 |
|  | F0 vs. F3 | Cu^-/+^ vs. Cu^-/+/-/-/-^ | 18,750 | 5,990 | 3,130 | 0,002 |
| Lysine methyltransferase 2E | F0 vs. F3 | Control vs. Cu^-/+/-/-/-^ | 19,250 | 5,990 | 3,214 | 0,001 |
|  | F3 vs. F3 | Cu^+/+/-/-/-^ vs. Cu^-/+/-/-/-^ | 22,139 | 5,821 | 3,803 | 0,000 |
|  | F0 vs. F3 | Cu^-/+^ vs. Cu^-/+/-/-/-^ | 20,375 | 5,990 | 3,402 | 0,001 |
| Eggless | F0 vs. F3 | Control vs. Cu^+/+/-/-/-^ | -13,986 | 5,821 | -2,403 | 0,016 |
|  | F0 vs. F3 | Control vs. Cu^-/+/-/-/-^ | 12,500 | 5,990 | 2,087 | 0,037 |
|  | F3 vs. F3 | Cu^+/+/-/-/-^ vs. Cu^-/+/-/-/-^ | 26,486 | 5,821 | 4,550 | 0,000 |
|  | F0 vs. F3 | Cu^-/+^ vs. Cu^-/+/-/-/-^ | 18,375 | 5,990 | 3,068 | 0,002 |
| Euchromatic histone lysine methyltransferase 1 | F0 vs. F3 | Control vs. Cu^+/+/-/-/-^ | -12,833 | 5,821 | -2,205 | 0,027 |
|  | F0 vs. F3 | Control vs. Cu^-/+/-/-/-^ | 13,250 | 5,990 | 2,212 | 0,027 |
|  | F3 vs. F3 | Cu^+/+/-/-/-^ vs. Cu^-/+/-/-/-^ | 26,083 | 5,821 | 4,481 | 0,000 |
|  | F0 vs. F3 | Cu^-/+^ vs. Cu^-/+/-/-/-^ | 21,500 | 5,990 | 3,590 | 0,000 |
| DOT1 like histone lysine methyltransferase | F0 vs. F3 | Control vs. Cu^-/+/-/-/-^ | 18,750 | 5,990 | 3,130 | 0,002 |
|  | F3 vs. F3 | Cu^+/+/-/-/-^ vs. Cu^-/+/-/-/-^ | 22,167 | 5,821 | 3,808 | 0,000 |
|  | F0 vs. F3 | Cu^-/+^ vs. Cu^-/+/-/-/-^ | 18,500 | 5,990 | 3,089 | 0,002 |
| Nuclear receptor binding SET domain protein 2 | F0 vs. F0 | Control vs. Cu^-/+^ | 12,875 | 5,990 | 2,150 | 0,032 |
|  | F0 vs. F3 | Control vs. Cu^+/+/-/-/-^ | -16,000 | 5,821 | -2,749 | 0,006 |
|  | F3 vs. F3 | Cu^+/+/-/-/-^ vs. Cu^-/+/-/-/-^ | 26,625 | 5,821 | 4,574 | 0,000 |
|  | F0 vs. F3 | Cu^-/+^ vs. Cu^-/+/-/-/-^ | 23,500 | 5,990 | 3,923 | 0,000 |
| SET domain containing 1A, histone lysine methyltransferase | F0 vs. F0 | Control vs. Cu^-/+^ | 12,250 | 5,990 | 2,045 | 0,041 |
|  | F0 vs. F3 | Control vs. Cu^+/+/-/-/-^ | -12,681 | 5,821 | -2,178 | 0,029 |
|  | F0 vs. F3 | Control vs. Cu^-/+/-/-/-^ | 13,250 | 5,990 | 2,212 | 0,027 |
|  | F3 vs. F3 | Cu^+/+/-/-/-^ vs. Cu^-/+/-/-/-^ | 25,931 | 5,821 | 4,455 | 0,000 |
|  | F0 vs. F3 | Cu^-/+^ vs. Cu^-/+/-/-/-^ | 25,500 | 5,990 | 4,257 | 0,000 |
| SET domain containing 2, histone lysine methyltransferase | F0 vs. F3 | Control vs. Cu^+/+/-/-/-^ | 17,236 | 5,821 | 2,961 | 0,003 |
|  | F0 vs. F3 | Control vs. Cu^-/+/-/-/-^ | 26,875 | 5,990 | 4,487 | 0,000 |
|  | F0 vs. F3 | Cu^-/+^ vs. Cu^-/+/-/-/-^ | 20,500 | 5,990 | 3,423 | 0,001 |
| SET And MYND domain containing 3 | F0 vs. F3 | Control vs. Cu^+/+/-/-/-^ | -14,403 | 5,821 | -2,474 | 0,013 |
|  | F0 vs. F3 | Control vs. Cu^-/+/-/-/-^ | 12,125 | 5,990 | 2,024 | 0,043 |
|  | F3 vs. F3 | Cu^+/+/-/-/-^ vs. Cu^-/+/-/-/-^ | 26,528 | 5,821 | 4,557 | 0,000 |
|  | F0 vs. F3 | Cu^-/+^ vs. Cu^-/+/-/-/-^ | 21,500 | 5,990 | 3,590 | 0,000 |
| Lysine methyltransferase 5B | F0 vs. F0 | Control vs. Cu^-/+^ | 12,875 | 5,990 | 2,150 | 0,032 |
|  | F0 vs. F3 | Control vs. Cu^+/+/-/-/-^ | -15,194 | 5,821 | -2,610 | 0,009 |
|  | F3 vs. F3 | Cu^+/+/-/-/-^ vs. Cu^-/+/-/-/-^ | 26,444 | 5,821 | 4,543 | 0,000 |
|  | F0 vs. F3 | Cu^-/+^ vs. Cu^-/+/-/-/-^ | 24,125 | 5,990 | 4,028 | 0,000 |
| Histone acetyltransferase 2A | F0 vs. F3 | Control vs. Cu^+/+/-/-/-^ | -15,750 | 5,821 | -2,706 | 0,007 |
|  | F3 vs. F3 | Cu^+/+/-/-/-^ vs. Cu^-/+/-/-/-^ | 27,250 | 5,821 | 4,681 | 0,000 |
|  | F0 vs. F3 | Cu^-/+^ vs. Cu^-/+/-/-/-^ | 22,000 | 5,990 | 3,673 | 0,000 |
| Lysine acetyltransferase 5 | F0 vs. F0 | Control vs. Cu^-/+^ | 12,000 | 5,990 | 2,003 | 0,045 |
|  | F0 vs. F3 | Control vs. Cu^+/+/-/-/-^ | -15,083 | 5,821 | -2,591 | 0,010 |
|  | F3 vs. F3 | Cu^+/+/-/-/-^ vs. Cu^-/+/-/-/-^ | 24,958 | 5,821 | 4,288 | 0,000 |
|  | F0 vs. F3 | Cu^-/+^ vs. Cu^-/+/-/-/-^ | 21,875 | 5,990 | 3,652 | 0,000 |
| Lysine acetyltransferase 6A | F0 vs. F3 | Control vs. Cu^+/+/-/-/-^ | -16,389 | 5,821 | -2,816 | 0,005 |
|  | F3 vs. F3 | Cu^+/+/-/-/-^ vs. Cu^-/+/-/-/-^ | 27,764 | 5,821 | 4,770 | 0,000 |
|  | F0 vs. F3 | Cu^-/+^ vs. Cu^-/+/-/-/-^ | 21,625 | 5,990 | 3,610 | 0,000 |
| Lysine acetyltransferase 7 | F0 vs. F0 | Control vs. Cu^-/+^ | 12,000 | 5,990 | 2,003 | 0,045 |
|  | F0 vs. F3 | Control vs. Cu^+/+/-/-/-^ | -13,583 | 5,821 | -2,334 | 0,020 |
|  | F0 vs. F3 | Control vs. Cu^-/+/-/-/-^ | 11,875 | 5,990 | 1,983 | 0,047 |
|  | F3 vs. F3 | Cu^+/+/-/-/-^ vs. Cu^-/+/-/-/-^ | 25,458 | 5,821 | 4,374 | 0,000 |
|  | F0 vs. F3 | Cu^-/+^ vs. Cu^-/+/-/-/-^ | 23,875 | 5,990 | 3,986 | 0,000 |
| Histone acetyltransferase 8 | F0 vs. F0 | Control vs. Cu^-/+^ | 14,625 | 5,990 | 2,442 | 0,015 |
|  | F0 vs. F3 | Control vs. Cu^+/+/-/-/-^ | -16,472 | 5,821 | -2,830 | 0,005 |
|  | F3 vs. F3 | Cu^+/+/-/-/-^ vs. Cu^-/+/-/-/-^ | 26,597 | 5,821 | 4,569 | 0,000 |
|  | F0 vs. F3 | Cu^-/+^ vs. Cu^-/+/-/-/-^ | 24,750 | 5,990 | 4,132 | 0,000 |
| Histone deacetylase complex subunit SAP18 | F0 vs. F3 | Control vs. Cu^-/+/-/-/-^ | 16,250 | 5,990 | 2,713 | 0,007 |
|  | F3 vs. F3 | Cu^+/+/-/-/-^ vs. Cu^-/+/-/-/-^ | 21,792 | 5,821 | 3,744 | 0,000 |
|  | F0 vs. F3 | Cu^-/+^ vs. Cu^-/+/-/-/-^ | 22,500 | 5,990 | 3,757 | 0,000 |
| DNA methyltransferase 1 | F0 vs. F0 | Control vs. Cu^-/+^ | 14,875 | 5,990 | 2,483 | 0,013 |
|  | F0 vs. F3 | Control vs. Cu^+/+/-/-/-^ | -16,875 | 5,821 | -2,899 | 0,004 |
|  | F3 vs. F3 | Cu^+/+/-/-/-^ vs. Cu^-/+/-/-/-^ | 25,125 | 5,821 | 4,316 | 0,000 |
|  | F0 vs. F3 | Cu^-/+^ vs. Cu^-/+/-/-/-^ | 23,125 | 5,990 | 3,861 | 0,000 |
| DNA methyltransferase 3 alpha | F0 vs. F3 | Control vs. Cu^-/+/-/-/-^ | 17,500 | 5,990 | 2,922 | 0,003 |
|  | F3 vs. F3 | Cu^+/+/-/-/-^ vs. Cu^-/+/-/-/-^ | 24,944 | 5,821 | 4,285 | 0,000 |
|  | F0 vs. F3 | Cu^-/+^ vs. Cu^-/+/-/-/-^ | 22,750 | 5,990 | 3,798 | 0,000 |
| RAD52 homolog, DNA repair protein | F0 vs. F3 | Control vs. Cu^+/+/-/-/-^ | -13,917 | 5,821 | -2,391 | 0,017 |
|  | F0 vs. F3 | Control vs. Cu^-/+/-/-/-^ | 12,625 | 5,990 | 2,108 | 0,035 |
|  | F3 vs. F3 | Cu^+/+/-/-/-^ vs. Cu^-/+/-/-/-^ | 26,542 | 5,821 | 4,560 | 0,000 |
|  | F0 vs. F3 | Cu^-/+^ vs. Cu^-/+/-/-/-^ | 22,125 | 5,990 | 3,694 | 0,000 |
| DNA repair protein RAD51 homolog | F0 vs. F3 | Control vs. Cu^+/+/-/-/-^ | -16,139 | 5,821 | -2,773 | 0,006 |
|  | F3 vs. F3 | Cu^+/+/-/-/-^ vs. Cu^-/+/-/-/-^ | 26,639 | 5,821 | 4,576 | 0,000 |
|  | F0 vs. F3 | Cu^-/+^ vs. Cu^-/+/-/-/-^ | 20,750 | 5,990 | 3,464 | 0,001 |
| Damage specific DNA binding protein 1 | F0 vs. F3 | Control vs. Cu^-/+/-/-/-^ | 15,375 | 5,990 | 2,567 | 0,010 |
|  | F3 vs. F3 | Cu^+/+/-/-/-^ vs. Cu^-/+/-/-/-^ | 22,444 | 5,821 | 3,856 | 0,000 |
|  | F0 vs. F3 | Cu^-/+^ vs. Cu^-/+/-/-/-^ | 24,250 | 5,990 | 4,049 | 0,000 |
| MRE11 homolog, double Strand break repair nuclease, meiotic recombination | F0 vs. F0 | Control vs. Cu^-/+^ | 14,625 | 5,990 | 2,442 | 0,015 |
|  | F0 vs. F3 | Control vs. Cu^+/+/-/-/-^ | -14,208 | 5,821 | -2,441 | 0,015 |
|  | F3 vs. F3 | Cu^+/+/-/-/-^ vs. Cu^-/+/-/-/-^ | 25,458 | 5,821 | 4,374 | 0,000 |
|  | F0 vs. F3 | Cu^-/+^ vs. Cu^-/+/-/-/-^ | 25,875 | 5,990 | 4,320 | 0,000 |
| X-ray repair cross complementing 1 | F0 vs. F3 | Control vs. Cu^-/+/-/-/-^ | 15,375 | 5,990 | 2,567 | 0,010 |
|  | F3 vs. F3 | Cu^+/+/-/-/-^ vs. Cu^-/+/-/-/-^ | 25,361 | 5,821 | 4,357 | 0,000 |
|  | F0 vs. F3 | Cu^-/+^ vs. Cu^-/+/-/-/-^ | 23,125 | 5,990 | 3,861 | 0,000 |
| Catalase | F0 vs. F3 | Control vs. Cu^-/+/-/-/-^ | 12,750 | 5,990 | 2,129 | 0,033 |
|  | F3 vs. F3 | Cu^+/+/-/-/-^ vs. Cu^-/+/-/-/-^ | 16,806 | 5,821 | 2,887 | 0,004 |
|  | F0 vs. F3 | Cu^-/+^ vs. Cu^-/+/-/-/-^ | 22,875 | 5,990 | 3,819 | 0,000 |
| Glutathion-S-transferase theta 1 | F0 vs. F3 | Control vs. Cu^+/+/-/-/-^ | 14,417 | 5,821 | 2,477 | 0,013 |
|  | F0 vs. F3 | Control vs. Cu^-/+/-/-/-^ | 22,375 | 5,990 | 3,736 | 0,000 |
|  | F0 vs. F3 | Cu^-/+^ vs. Cu^-/+/-/-/-^ | 21,625 | 5,990 | 3,610 | 0,000 |
| Glutathion-S-transferase theta 5 | F0 vs. F3 | Control vs. Cu^-/+/-/-/-^ | 13,000 | 5,990 | 2,170 | 0,030 |
|  | F0 vs. F3 | Cu^-/+^ vs. Cu^-/+/-/-/-^ | 24,500 | 5,990 | 4,090 | 0,000 |
| Glutathion-S-transferase theta | F0 vs. F0 | Control vs. Cu^+/+^ | 12,500 | 5,990 | 2,087 | 0,037 |
|  | F0 vs. F0 | Control vs. Cu^-/+^ | 13,750 | 5,990 | 2,296 | 0,022 |
|  | F3 vs. F3 | Cu^+/+/-/-/-^ vs. Cu^-/+/-/-/-^ | 21,417 | 5,821 | 3,679 | 0,000 |
|  | F0 vs. F3 | Cu^-/+^ vs. Cu^-/+/-/-/-^ | 23,875 | 5,990 | 3,986 | 0,000 |

**Table S3.** Table representing increased and decreased expression (green and ↑↑↑ vs. red and **↓↓↓** cells, respectively) of genes in different treatments (Treat.) compared to the references (Ref.). Only genes found to be significantly differentially expressed (Kruskal-Wallis; p-value < 0.05) are represented. Gene and treatment-specific mRNA levels are interpreted in manuscript Section 3.2.

|  | **Ref.** | **Control** | | | | **Cu^+/+^** | | **Cu^-/+^** | **Cu^+/+/-/-/-^** |
| --- | --- | --- | --- | --- | --- | --- | --- | --- | --- |
| **GENE** | **Treat.** | **Cu^+/+^** | **Cu^-/+^** | **Cu^+/+/-/-/-^** | **Cu^-/+/-/-/-^** | **Cu^-/+^** | **Cu^+/+/-/-/-^** | **Cu^-/+/-/-/-^** | **Cu^-/+/-/-/-^** |
| ***Clock*** |  |  | **↑↑↑** |  | **↓↓↓** |  |  | **↓↓↓** | **↓↓↓** |
| ***TIPIN*** |  | **↓↓↓** |  | **↓↓↓** |  | **↓↓↓** | **↓↓↓** |  | **↑↑↑** |
| ***Crypt1*** |  |  |  | **↑↑↑** | **↓↓↓** |  | **↑↑↑** | **↓↓↓** | **↓↓↓** |
| ***PIWI*** |  |  |  | **↑↑↑** |  |  | **↑↑↑** | **↓↓↓** | **↓↓↓** |
| ***Arg1*** |  |  | **↑↑↑** | **↑↑↑** | **↓↓↓** |  |  | **↓↓↓** | **↓↓↓** |
| ***Arg2*** |  |  | **↑↑↑** |  | **↓↓↓** |  |  | **↓↓↓** | **↓↓↓** |
| ***DICER*** |  |  |  | **↑↑↑** | **↓↓↓** |  |  | **↓↓↓** | **↓↓↓** |
| ***HDAC1*** |  |  |  | **↑↑↑** |  |  |  | **↓↓↓** | **↓↓↓** |
| ***HDAC3*** |  |  |  |  | **↓↓↓** |  |  | **↓↓↓** | **↓↓↓** |
| ***HDAC4*** |  |  |  |  | **↓↓↓** |  |  | **↓↓↓** | **↓↓↓** |
| ***HDAC6*** |  |  |  | **↑↑↑** | **↓↓↓** |  |  | **↓↓↓** | **↓↓↓** |
| ***HDAC8*** |  |  |  | **↑↑↑** | **↓↓↓** |  |  | **↓↓↓** | **↓↓↓** |
| ***KMT2A*** |  |  | **↑↑↑** | **↑↑↑** | **↓↓↓** | **↑↑↑** | **↑↑↑** | **↓↓↓** | **↓↓↓** |
| ***KMT2C*** |  | **↓↓↓** |  | **↓↓↓** | **↓↓↓** |  |  | **↓↓↓** |  |
| ***KMT2E*** |  |  |  |  | **↓↓↓** |  |  | **↓↓↓** | **↓↓↓** |
| ***Eggless*** |  |  |  | **↑↑↑** | **↓↓↓** |  |  | **↓↓↓** | **↓↓↓** |
| ***EHMT1*** |  |  |  | **↑↑↑** | **↓↓↓** |  |  | **↓↓↓** | **↓↓↓** |
| ***DOT1L*** |  |  |  |  | **↓↓↓** |  |  | **↓↓↓** | **↓↓↓** |
| ***NSD2*** |  |  | ↑↑↑ | **↑↑↑** |  |  |  | **↓↓↓** | **↓↓↓** |
| ***SEDT1*** |  |  | ↑↑↑ | **↑↑↑** | **↓↓↓** |  |  | **↓↓↓** | **↓↓↓** |
| ***SEDT2*** |  |  |  | **↓↓↓** | **↓↓↓** |  |  | **↓↓↓** |  |
| ***SMYD3*** |  |  |  | **↑↑↑** | **↓↓↓** |  |  | **↓↓↓** | **↓↓↓** |
| ***KMT5B*** |  |  | ↑↑↑ | **↑↑↑** |  |  |  | **↓↓↓** | **↓↓↓** |
| ***KAT2A*** |  |  |  | **↑↑↑** |  |  |  | **↓↓↓** | **↓↓↓** |
| ***KAT5*** |  |  | ↑↑↑ | **↑↑↑** |  |  |  | **↓↓↓** | **↓↓↓** |
| ***KAT6A*** |  |  |  | **↑↑↑** |  |  |  | **↓↓↓** | **↓↓↓** |
| ***KAT7*** |  |  | ↑↑↑ | **↑↑↑** | **↓↓↓** |  |  | **↓↓↓** | **↓↓↓** |
| ***KAT8*** |  |  | ↑↑↑ | **↑↑↑** |  |  |  | **↓↓↓** | **↓↓↓** |
| ***SAP18*** |  |  |  |  | **↓↓↓** |  |  | **↓↓↓** | **↓↓↓** |
| ***DNMT1*** |  |  | ↑↑↑ | **↑↑↑** |  |  |  | **↓↓↓** | **↓↓↓** |
| ***DNMT3A*** |  |  |  |  | **↓↓↓** |  |  | **↓↓↓** | **↓↓↓** |
| ***RAD52*** |  |  |  | **↑↑↑** | **↓↓↓** |  |  | **↓↓↓** | **↓↓↓** |
| ***RAD51*** |  |  |  | **↑↑↑** |  |  |  | **↓↓↓** | **↓↓↓** |
| ***DDB1*** |  |  |  |  | **↓↓↓** |  |  | **↓↓↓** | **↓↓↓** |
| ***MRE11*** |  |  | **↑↑↑** | **↑↑↑** |  |  |  | **↓↓↓** | **↓↓↓** |
| ***XRCC1*** |  |  |  |  | **↓↓↓** |  |  | **↓↓↓** | **↓↓↓** |
| ***CAT*** |  |  |  |  | **↓↓↓** |  |  | **↓↓↓** | **↓↓↓** |
| ***GST1*** |  |  |  | **↓↓↓** | **↓↓↓** |  |  | **↓↓↓** |  |
| ***GST5*** |  |  |  |  | **↓↓↓** |  |  | **↓↓↓** |  |
| ***GSTt*** |  | **↑↑↑** | **↑↑↑** |  |  |  |  | **↓↓↓** | **↓↓↓** |
